# The use of malignant serosal fluid as a tumor vaccine in patients with advance malignancies

**DOI:** 10.1186/2051-1426-3-S2-P439

**Published:** 2015-11-04

**Authors:** Suresh Katakkar

**Affiliations:** 1 Arizona Hematology Oncology, Tucson, AZ, USA

## 

After getting approval from IRB 15 patients were treated with the vaccine prepared from their own malignant serosal fluid. This study was done 1990. These were patients who had all conventional chemotherapy and have failed and had progressive disease. The serosal fluid was heat treated under aseptic condition so as to maintain the antigenecity but to lose the virulence of the malignant cells. The fluid then was injected subcutaneously half cc QOD first week and twice week second week and then once week. The patients were informed to report any unusual symptoms such as fever, rash or swelling at the injection site or any systemic effects. The patients were examined every month by the physician and the appropriate radiological work up was done every three months or earlier if needed. All the laboratory work was done to begin with and every fourth week.

## Results

One patient with advanced gastric cancer had CR for five years, 2/4 NSCLC had partial response for 6 months,1/4 breast cancer had PR for 5.8 months,2/3 ovarian cancer had PR for 7.8 months and 1/2 colon cancer had PR for 4.5 months and 1/1 pancreatic cancer had PR for 4 months. No adverse events were noted other than mild itching in 4 patients at the site of injection. The PR was consider if there was at least 25% reduction in the metastatic disease. The patient with CR lived for 5 and half years and had died at the age of 95.

## Methodology

The fluid was heated at 37 degree centigrade for 45 minutes in aliquots of 5cc test tubes in a water bath. Post heat treatment was analyzed for malignant cells. The culture and sensitivity was done to make sure there no was contamination. The study on the fluid was limited as the technology was not advanced then. But now we can determine Exons, cytokines or genetic analysis, microRNAs.

## Conclusion

This initial crude method of making the vaccine was successful in getting response 8/15 patients, 1 patient had very long-term response excluding her PR average of 4 months without any side effects. Now the technology has advanced so we may be able to isolate what antigenic molecule we are injecting or is it the entire cytoskeleton that is needed. Besides the patient who had CR had gastric cancer and as we now know the cancer genomics is more unique in gastric cancers such personalized therapy may be more specific in gastric cancer.

